# RETRACTION: Phytomedicinal Role of *Pithecellobium dulce* against CCl_4_-mediated Hepatic Oxidative Impairments and Necrotic Cell Death

**DOI:** 10.1155/ecam/9817496

**Published:** 2025-11-10

RETRACTION: P. Manna, S. Bhattacharyya, J. Das, J. Ghosh, and P. C. Sil, “Phytomedicinal Role of *Pithecellobium dulce* against CCl_4_-Mediated Hepatic Oxidative Impairments and Necrotic Cell Death,” *Evidence-Based Complementary and Alternative Medicine*, vol. 2011 (2011), https://doi.org/10.1093/ecam/neq065.

The above article, published online on 13 March 2011 in Wiley Online Library (https://wileyonlinelibrary.com), has been retracted by John Wiley & Sons Ltd. The retraction has been agreed following an investigation of the issues raised by *Lima fujitai* and *Hoya Camphorifolia* on PubPeer [1], which highlighted multiple concerns with the figures as follows:

- The fluorescence image of CCl_4_ treated mice in Figure 4a appears in a number of other publications [2–6] by the same author group, where it is presented as part of different experiments.
- The liver tissue shown in panels a and b of Figure 9 appears identical despite representing different treatment groups. Further investigation has also identified identical features between panels c and f.

As a result of this investigation, the results of the article are no longer considered reliable.

The authors disagree with this retraction.
